# Serological Prevalence of *Toxoplasma gondii*, *Neospora caninum*, and *Sarcoptes scabiei* var. *suis* in Wild Boars (*Sus scrofa*) Hunted in a Highly Anthropized Area in Italy

**DOI:** 10.3390/ani13111730

**Published:** 2023-05-23

**Authors:** Luca Villa, Carolina Allievi, Alessia Libera Gazzonis, Giordano Ventura, Matteo Gradassi, Sergio Aurelio Zanzani, Maria Teresa Manfredi

**Affiliations:** 1 Department of Veterinary Medicine and Animal Sciences, Università degli Studi di Milano, Via dell’Università 6, 26900 Lodi, Italy; carolina.allievi@unimi.it (C.A.); alessia.gazzonis@unimi.it (A.L.G.); sergio.zanzani@unimi.it (S.A.Z.); 2 Istituto Zooprofilattico Sperimentale della Lombardia e dell’Emilia-Romagna “Bruno Ubertini”, Via Cardinal Massaia 7, 26100 Cremona, Italy; giordano.ventura@izsler.it (G.V.); matteo.gradassi@izsler.it (M.G.)

**Keywords:** *Toxoplasma gondii*, *Neospora caninum*, *Sarcoptes scabiei*, wild boars, zoonosis

## Abstract

**Simple Summary:**

In recent years, wild boars’ populations have been expanding both in rural and urban areas in Europe. Since these animals are placed in the interface of domestic and sylvatic cycle for zoonotic and animal-specific parasites, the aim of this study was to evaluate the serological prevalence of *Toxoplasma gondii*, *Neospora caninum*, and *Sarcoptes scabiei* var. *suis* in wild boars hunted in an anthropized area in Italy. The results confirmed the possible risk of *T. gondii* infection for humans from consumption of meat and meat products from wild boars. Besides, their role as intermediate hosts for *N. caninum* is relevant due to the high presence of dairy cattle farms in the area. The usefulness of serological analysis to estimate *S. scabiei* infestation in wild boars is confirmed to evaluate the sanitary risk for livestock and humans in the area. In conclusion, the constitution of surveillance plans by standardized methods should be emphasized also to promote the awareness among hunters, wildlife professionals, and consumers on the circulation of parasites of this wild species with impact both on human and animal health.

**Abstract:**

Due to the increasing expansion into urban and rural areas, wild boars represent a potential source of infection with zoonotic and animal-specific parasites for both humans and animals. Therefore, the aim of this study was to investigate the serological prevalence to *Toxoplasma gondii*, *Neospora caninum*, and *Sarcoptes scabiei* var. *suis* in blood samples from wild boars (*Sus scrofa*) hunted in an anthropized area in Italy. Enzyme-linked immunosorbent assay (ELISA) tests were used to detect antibodies anti-*T. gondii* and anti-*S. scabiei* and an immunofluorescence antibody test (IFAT) for antibodies anti-*N. caninum*. 81 out of 128 wild boars (P = 63.3%) resulted positive for at least one of the three parasites. 68 of them were seropositive to *T. gondii* (P = 53.1%) and 14 to *N. caninum* (P = 10.9%). 9 wild boars resulted seropositive to *S. scabiei* var. *suis* (P = 7.0%). Sampling season was the only significant risk factor related to *S. scabiei* var. *suis* seroprevalence (OR = 7.8). The high occurrence of *T. gondii* supports the role of this species as a source of infection for other animals and humans. Furthermore, the serological prevalence of *N. caninum* and *S. scabiei* var. *suis* in wild boars from the study area characterized by the presence of numerous dairy cattle and pig farms is relevant to demonstrate its suitability for the circulation of these parasites both in domestic and wild species.

## 1. Introduction

In recent years wild boars (*Sus scrofa*) have been increasingly expanding in terms of both the number of animals and the range of habitat. The variation in the geographical distribution of this wild species is mainly caused by climate change and human activities, such as the abandonment of fields, the reduction in the number of extensive livestock farms, the rise in forest cover, and the expansion of cities into semi-urban environments close to rural areas [1]. Apart from the ecological impact and the conflict with human activities, the increased frequency of contacts among wild species, livestock, and humans, and the increased human consumption of game meat, may lead to the transmission of pathogens. Indeed, wild boars are reservoirs of bacteria, viruses, and parasites, representing a source of infection for both humans and domestic animals [2,3].

Among parasites, *T. gondii* and *N. caninum* (Apicomplexa, and Sarcocystidae) are two morphologically similar obligated intracellular cyst-forming protozoa with a wild cycle closely interfacing with the domestic cycle [4,5]. *Toxoplasma gondii* is a ubiquitous zoonotic protozoan with an indirect life cycle involving domestic and wild felines as the definitive hosts and mammals and birds as the intermediate hosts [5]. Toxoplasmosis is one of the most common parasitic zoonoses worldwide, with approximately 30% of people infected with *T. gondii* [6]. Parasite infection can have severe consequences in immunocompromised people, children, and pregnant women. In recent years, the increased consumption of game meat in Europe due to the rise in hunted wild boars [1] is considered an emerging risk factor for the transmission of food-borne pathogens, including *T. gondii* infection in humans [7,8]. Game meat is usually consumed well-cooked, reaching the target temperature for tissue cyst inactivation; however, in some culinary traditions, the consumption of raw or undercooked meat and cured meats is a risk for parasite transmission [3,7,9,10]. Toxoplasmosis is also considered an occupational disease for butchers and hunters exposed to the infection due to meat handling [3,7]. In Europe, a pooled seroprevalence of 26% was reported in wild boars [11]; in Italy, seroprevalence values ranged between 12.2 and 43.3% [12,13,14,15,16].

*Neospora caninum* is the causative agent of neosporosis, a severe clinical disease of cattle and dogs worldwide [17]. Indeed, the parasite is a major cause of abortion in cattle with huge economic losses to the dairy and beef industries [18]. Domestic dogs and wild canids are the definitive hosts; ruminants, equids, and swine can act as intermediate hosts of the parasite [19,20,21,22]. Serological evidence in wild animals indicates that many species, including wild boars, were infected [23]. Serological studies on the spread of *N. caninum* were conducted in wild boars worldwide, including some European countries [24,25,26], demonstrating the infection of this wild species. In humans, despite some serological evidence, infection has not been confirmed, and, therefore, to date, *N. caninum* is not considered a zoonotic parasite [27]. In Italy, no serological data for wild boars are available. More recently, molecular analysis of wild boar fetuses from animals hunted in north-western Italy seems to support a congenital transmission of *N. caninum* in this host [28].

*Sarcoptes scabiei* (Arthropoda, and Sarcoptidae), the burrowing mite, includes a complex of varieties of the single species, causing sarcoptic mange in more than 150 mammals species worldwide [29]. Furthermore, this mite is responsible for scabies in humans, a fact which was recently included as part of the World Health Organization (WHO) roadmap for neglected tropical diseases 2021–2030 [30]. Transmission between hosts occurs primarily through prolonged direct contact with an infested host; however, since *Sarcoptes* mites can survive off their hosts for some time, depending on ambient relative humidity and temperature, infestation by indirect contact (via fomites) is also possible [29]. Typical clinical signs are itching and a pimple-like skin rash in various areas of the body with different localization according to animal species. In humans, the varieties of animal origin are responsible for zoonotic scabies, or “pseudoscabies”, considered a self-limiting disease with a short incubation period and transient clinical signs limited to some topographic regions of the body [31]. Sarcoptic mange is responsible for significant morbidity and mortality in both livestock and wildlife, including wild boars [31,32,33]. Scavenger habits could expose wild boars to an increased risk of infestation; another risk factor is represented by the interactions between wild boars and domestic pigs [31]. Recent serosurveys suggested that *S. scabiei* is widely distributed in free-ranging wild boars in France, Sweden, Italy, and Spain [32,34].

Since wild boars are perfectly placed in the interface of domestic and sylvatic cycle as a potential source of infection of several zoonotic and animal-specific parasites both for humans and other animals, the aim of this study was to evaluate the serological prevalence of selected parasites, i.e., *T. gondii*, *N. caninum*, and *S. scabiei* var. *suis*, in wild boars in the province of Cremona (Lombardy, Italy) and to evaluate the risk factors that could have a predictive value for a seropositive outcome. In this way, it was also possible to assess the role of this ungulate species as a bioindicator of the circulation of zoonotic and animal parasites in a highly anthropized area also characterized by a high number of livestock farms.

## 2. Materials and Methods

### 2.1. Study Area

Wild boars were hunted in an area in the centre of the Po Valley (45°08′ N 10°02′ E) comprising 12 municipalities (Cremona province, Lombardy region), characterized by a flat territory with an average altitude of 45 m above sea level (m a.s.l.), also surrounded by several large rivers (Adda, Po, Serio, and Tormo). The area, suited to intensive agriculture and animal breeding, is one of the largest livestock production areas in Italy and stands out for the high density of farms, mainly based on the intensive production system. In particular, in the province of Cremona, the Italian National Zootechnical Registry counted 1221 cattle farms (366 beef, 792 dairy, and 62 mixed) hosting 309,827 animals, and 495 swine farms hosting 923,983 animals (National Zootechnical Database, https://www.vetinfo.sanita.it; accessed on 21 March 2023). Due to the increase of wild boars’ populations, these ungulates were reported recently in this area, particularly near the Po River. The climate of the study area is continental, with hot muggy summers and cold and foggy winters. A large annual thermal excursion is recorded with a mean maximum temperature of 25–28 °C and a mean minimum temperature of −1 to −2 °C. Relative humidity values are quite high throughout the year, from a minimum of 67.1% to a maximum of 100%. Rainfall is distributed over the year; the average annual precipitation is around 750 mm with a peak in the autumn season.

### 2.2. Study Population, and Sample and Data Collection

During a one-year period from March 2019 to March 2020, 128 wild boars regularly hunted within the regional wildlife surveillance program and, destined for human consumption, were sampled.

Sampling was performed during the mandatory inspection of carcasses at the game-handling establishment in collaboration with the Istituto Zooprofilattico Sperimentale della Lombardia e dell’Emilia-Romagna “Bruno Ubertini” (Cremona, Italy). Blood samples were taken from the heart, freshly open blood vessels, or body cavities from each animal. After collection, sera were separated by centrifugation (2120× *g*, 15 min) and then stored at −20 °C until serological analyses.

For each animal, individual data (age and sex), sampling date, and hunting municipality were collected. The animals were divided into three age classes according to the monitoring and control plan for wildlife of Lombardy region: young (<1 year old), subadult (1–2 years old) and adult (>2 years old). Besides, wild boars were classified according to the sampling date in two seasons, i.e., spring–summer from March to August and autumn–winter from September to February. Concerning hunting municipality, data on number of inhabitants, surface, and human density were noted for each and they were categorized as rural (<100 inh/km^2^), intermediate (100–499 inh/km^2^), and urban (>500 inh/km^2^) [35].

### 2.3. Serological Analyses

Serum samples were analysed for *T. gondii* using an indirect ELISA kit (ID Screen^®^ Toxoplasmosis Indirect Multi-species, Innovative Diagnostics, France), validated for the detection of anti-*T. gondii* antibodies in sera from several species, including wild boars [15].

Moreover, *N. caninum* antibodies were detected using an indirect immunofluorescence antibody test (IFAT), with slides coated with *N. caninum* antigens provided in a commercial kit (MegaScreen^®^ Fluo Neospora caninum, Megacor, Austria), following the manufacturer’s instructions, with slight modifications, according to Villa et al. [22]. An initial screening dilution of 1:50 was used; then, seropositive samples were two-fold serially diluted to determine the end-point antibody titer.

Finally, for the detection of anti-*S. scabiei* var. *suis* antibodies, sera were analysed with a commercial indirect ELISA kit (Sarcoptes-ELISA 2001^®^ Pig, Afosa GmbH, Germany), using *Sarcoptes* mites from pigs as antigen and validated for wild boars, following the manufacturer’s instructions [33].

### 2.4. Statistical Analysis

Seroprevalence of *T. gondii*, *N. caninum*, and *S. scabiei* var. *suis* in wild boars was calculated according to the considered categories, i.e., age class, sex, sampling season, and hunting municipality. Separate generalized linear models (GLMs) with binomial distribution were performed to verify the influence of individual data, sampling season, and hunting municipality on seropositivity to the selected parasites. The following factors, i.e., age class (young, subadult, and adult), sex (male and female), sampling season (spring–summer and autumn–winter), and hunting municipality (rural, intermediate, and urban), were entered in each model as explanatory variables. The binary outcome (negativity/positivity to *T. gondii*, *N. caninum*, and *S. scabiei* var. *suis*) based on serological analysis results was used as the response variable. The models were developed through a backward selection procedure (significance level to remove variables from the model = 0.05), based on Akaike information criterion (AIC) values. Statistical analysis was performed using SPSS software (Statistical Package for Social Science, IBM SPSS Statistics for Windows, 25.0, Chicago, IL, USA).

## 3. Results

Overall, 128 wild boars, 64 males and 64 females, were included in the study; these animals belonged to three age classes: young (*n* = 20), subadults (*n* = 56), and adults (*n* = 52). Regarding the sampling season, 44 animals were sampled in spring–summer and 84 in autumn–winter. According to the human density of the study area, 47 and 81 wild boars were hunted in rural and in intermediate municipalities, respectively.

81 wild boars (P = 63.3%) resulted positive for at least one of the three selected parasites. Serological results for *T. gondii*, *N. caninum*, and *S. scabiei* are summarized in Table 1.

In particular, 68 of them were seropositive to *T. gondii*, with a prevalence of 53.1%. The seroprevalence was equal to 7.8%, 24.2%, and 21.1% in young, subadult, and adult wild boars, respectively. Similar seroprevalence was recorded in male (P = 27.3%) and female (P = 25.8%) exemplars. The seroprevalence to *T. gondii* was higher in autumn–winter (P = 33.6%) than in spring–summer (P = 19.5%). Higher seropositivity values were also evidenced in the urban municipalities with an intermediate level of human density (P = 32.0%) than in the rural ones (P = 21.1%).

Anti-*N. caninum* antibodies were detected in 14 wild boars with a prevalence of 10.9%; three of them showed an antibody titer of 1:100 and the remaining were positive at the cut-off of 1:50. The seroprevalence ranged from 0.8% in young wild boars up to 3.9% and 6.2% in subadult and adult ones, respectively. Seroprevalence was similar considering gender with values of 6.2% in male and 4.7% in female animals. A higher positivity to *N. caninum* antibodies was revealed in autumn–winter (P = 9.4%) if compared to spring–summer (P = 1.6%). Seropositive animals were mostly hunted in intermediate urban areas (P = 8.6%) than in rural ones (P = 2.3%).

Nine wild boars resulted positive to *S. scabiei* var. *suis* antibodies with a seroprevalence of 7.0%. A higher seropositivity value was evidenced in subadults (P = 4.7%) if compared to young (P = 0.8%) and adult (P = 1.6%) wild boars and also in females than in male animals (P = 4.7% and 2.3%, respectively). A higher seroprevalence was also revealed in spring–summer (P = 5.5%) than in autumn–winter hunted wild boars (P = 1.6%). In relation to hunting territories, positive animals were mostly detected in intermediate (P = 5.5%) than rural municipalities (P = 1.6%).

For what concerns co-infections, the most common one was observed in six wild boars (P = 4.7%) between *T. gondii* and *N. caninum*. Besides, three wild boars (P = 2.3%) were seropositive to both *T. gondii* and *S. scabiei* var. *suis* and only one (P = 0.8%) to *N. caninum* and *S. scabiei* var. *suis*. None of the sampled animals resulted seropositive to all three investigated parasites.

By statistical analysis, only the variable “sampling season” was significantly associated with *S. scabiei* var. *suis* infestation and was entered in the final multivariate model. Indeed, wild boars hunted in spring–summer were at a higher risk of mite infestation than those hunted in autumn–winter (Table 2). For none of the other selected parasites, any association between seropositivity and the considered risk factors was not evidenced.

## 4. Discussion

This study contributes to update data on the seroprevalence of three selected parasites with impact on both human and animal health, i.e., *T. gondii*, *N. caninum*, and *S. scabiei* var. *suis*, in wild boars from an anthropized area in Italy. A high seroprevalence was evidenced with 63.3% of wild boars positive for at least one of the investigated parasites.

### 4.1. T. gondii Infection in Wild Boars’ Populations, Risk Factors, and Zoonotic Impact

Regarding *T. gondii*, an overall seroprevalence of 53.1% was reported. Serological diagnosis of *T. gondii* infection take advantage of the persistence of specific antibodies in serum following exposure to the parasite; among available tests ELISA appears to be the most reliable, practical, economical, and widely used test for the detection of exposure to *T. gondii* in both domestic and wild animals [11,15]. The commercial ELISA kit of this study was widely applied in other serosurveys both in livestock and in wildlife demonstrating good performances on swine samples (Se = 78–100%, and Sp = 98.25–100%). A lower result (P = 43.3%) was recorded in a previous study in northern Italy using the same diagnostic technique [15]; even if there is not a strong difference between these values, it should be considered that wild boars from this study were hunted in a highly anthropized area, while in the other survey wild boars were from a natural park where the circulation of *T. gondii* could be lower. Indeed, the human spatial dispersion acts as an influential factor in *T. gondii* infection prevalence in wild boars and the higher seroprevalence values could be found in animals in proximity of inhabited area [35,36]. Other Italian serosurveys in central and southern regions showed lower seroprevalence values between 12.2% [16] and 14% [12,14]. In Europe, a wide variation of the observed prevalence values of *T. gondii* in wild boars was reported using ELISA as diagnostic technique from less than 10% [37] to more than 40% in different countries [38,39]. In general, the heterogeneity of the seroprevalences may be caused by the diverse sample size and the investigated geographical area but also by the use of different serological methods and related cut-offs and the lack of standardization [12,40]. No significative difference in seropositivity was recorded with regard to age, sex, sampling season, and hunting municipality. As to age, some reports confirmed a stability to the parasite infection over the years [41,42]; however, an association between seroprevalence and age with an increase in the risk of infection in older animals was recorded [12,35,39,43]. Besides, concerning the sex of the animals, also previous studies evidenced similar seroprevalence values in males and females [15,44]. Any significant difference in the seropositivity to *T. gondii* was observed among wild boars from rural or intermediate hunting municipalities even if some studies recognized differences depending on the sampling area [45,46]. Wild boars are responsible for maintaining the wild cycle of *T. gondii* due to the multiple routes of infection and their highly variable diet; indeed, being omnivorous, these hosts become infected by ingesting food or drinking water contaminated by sporulated oocysts shed from cats or tissues containing cysts of other intermediate hosts [11,12,36]. Consequently, the presence of infected preys in the environment could assume a central role in the risk of infection [45,47]. For all these reasons and for the increased sharing of the same natural habitat with domestic animals, the assessment of *T. gondii* seroprevalence in wild boars may be an excellent indicator of the environmental contamination and the risk of transmission to domestic hosts [8,12,47,48,49,50,51].

In the view of the One Health approach, the presence of tissue cysts in the meat of wild boars and their long survival in muscle is a possible source of toxoplasmosis for humans [52]. Besides, these animals may also be reservoirs of other zoonotic pathogens [53,54]. Hunters and their families appear to be particularly exposed for their high consumption of this type of meat and for handling the meat during evisceration [8]. As there are currently no methods for identification and subsequent control of *T. gondii* during *post-mortem* inspection of carcasses, the European Food Safety Authority (EFSA) stressed the importance of monitoring this protozoan in game animals, defining it as a “high priority” and proposed the implementation of an integrated control and a prevention system covering the entire food chain “from farm to fork” [48,55]. However, it should be considered that the association between seropositivity and meat infectivity is not always demonstrated and in a seronegative animal it is not possible to rule out the presence of tissue cysts [8,52,55]. Therefore, further molecular studies to detect parasitic DNA and identify the *T. gondii* genotypes involved in the infection are needed, also considering that the severity of clinical toxoplasmosis in humans may vary depending on the virulence of the strain [15,40].

### 4.2. N. caninum Infection in Wild Boars’ Populations and Risks for Livestock

As concerns *N. caninum*, a seroprevalence of 10.9% was revealed, with three wild boars showing an antibody titer of 1:100 and the remaining nine animals positive at the cut-off of 1:50. Even if no significant differences in seropositivity were detected considering age, sex, season, or hunting municipality, seroprevalence values were higher in older animals, while similar exposure was evidenced between males and females. In particular, the higher prevalence in adult animals may be indicative of an increased risk of infection due to rooting behaviours and feeding on intermediate hosts of the parasite [56]. Serum samples were analyses by immunofluorescence antibody test previously used to detect anti-*N. caninum* antibodies in pigs [22]. The interpretation of the slides was performed comparing each well to the fluorescence of the positive and negative controls, considered as a reference pattern, and only a bright, sharp, and clear, yellow–green fluorescence on the membrane extending to the whole body of *N. caninum* tachyzoites was considered a positive reaction. Negative and positive control sera from both cattle and pigs were inserted. Since the immunofluorescence antibody test is very specific, it is usually used as confirmatory test; however, the validation of diagnostic test kits is still a challenge for wildlife. Only few data are available on the circulation of *N. caninum* in wild boar worldwide. Seropositivity values using the same diagnostic technique, applied in the current survey, were reported in Spain (0.3%), in the Czech Republic (10.3%), and in Brazil (10.8%) [24,25,57]. Wild boars root in the ground in search of food and feed on plant materials, live animals, and carcasses. This attitude increases the risk of infection with *N. caninum;* indeed, wild boars could be infected by ingesting food or drinking water contaminated by sporulated oocysts shed from the definitive hosts (e.g., dogs) or by tissues containing cysts of other intermediate hosts (e.g., micromammals) [25]. Besides, the parasite may be transmitted trans-placentally (congenital vertical transmission) from an infected sow to the foetuses during pregnancy, as demonstrated in domestic pigs experimentally infected and in wild boars with natural infection [28,58,59]. Moreover, the presence of wild boars was frequently signalled in proximity to cattle farms in which the circulation of *N. caninum* was previously demonstrated [60,61]. In such context, wild boars could easily be infected and become a source of infection for dogs feeding on infected wild boar carcasses helping to maintain *N. caninum* circulation in cattle farms [25,57,62]. Besides, *N. caninum* seems widespread in the wildlife being reported in wild ruminants [63], rodents [64], eastern cottontail rabbits [65], and wild birds [66,67]. Nevertheless, the role of wild boars in the epidemiology and maintenance of the wild cycle of *N. caninum* is still not well defined and further investigations are needed.

### 4.3. S. scabiei in Wildlife and Related Risks for Livestock and Humans

With regard to *S. scabiei* var. *suis*, 7.03% of wild boars showed seropositivity for the mite and sampling season was a risk factor associate to the positivity, with wild boars sampled in spring–summer more positive to *S. scabiei* if compared to those sampled in autumn–winter, probably due to climate, host behaviour, and endocrine activity [68]. Moreover, even if non-statistically-significant differences were detected, younger wild boars and females seem more exposed to mite infestation. In this study, the analyses were performed using a commercial ELISA kit to detect the presence of IgG antibodies against *S. scabiei* var. *suis* in samples of swine serum, also successfully validated and applied to wild boar sera with good performances (Se = 75% and Sp = 80%), even if lower than those indicated by the manufacturer for domestic pigs (Se = 94% and Sp = 97%). Previous studies on circulation and monitoring of this ectoparasite in wild populations are fragmentary. In a recent serological survey concerning wild boars from five different European countries (France, Italy, Spain, Sweden, and Switzerland), a range of prevalence from 0% to 17.4% was recorded. Particularly, in north-western Italy, a seroprevalence between 5.6 and 9.4% was evidenced, similar to our result [34]. Transmission of sarcoptic mange occurs primarily through direct contact between animals. However, indirect transmission between different host species is also possible; besides, animals with scavenger attitudes, including wild boars, may be at a higher risk of mite infestation [31,69,70]. Sarcoptic mange has been studied extensively in domestic pig farming due to the negative impact on productivity [71], whereas it has been poorly investigated in wild boars. In this regard, it should also be considered that even if the possible risk of interspecific transmission is underestimated, spill-over events were reported, and wild boars could represent a connection between wild and domestic parasite cycle [69]. Moreover, cases of transmission of this mite from wildlife to humans were described, with one single case from wild boar to human, also emphasizing the zoonotic risk [31]. Therefore, it is urgent to raise awareness among hunters, wildlife professionals, and veterinarians about *S. scabiei* var. *suis* infestation in wild populations and monitor the occurrence of this mite that may be distributed over a larger area than inferred when considering only wild boar with clinical signs of mange. For this reason, in wildlife, the serological approach could be a useful tool to diagnose also sub-clinical or chronic infections not detected by traditional diagnostic methods.

## 5. Conclusions

The seroprevalence values of the surveyed parasites in wild boars confirm the role of this species as a potential source of parasites for humans and animals. The feeding behaviour and the scavenging activity of this animal species are the main factors that could support this role. Moreover, other factors depending on human habits and the features of the study area should be considered. In particular, this area, highly anthropized and characterized by the presence of numerous cattle and pig farms with a very large number of animals confined, has been proved to be suitable for the circulation of parasites, not only among the domestic species, but also among the wild ones.

A high seropositivity to *T. gondii* confirms the possible risk for humans from consumption of meat and meat products from wild boars. Besides, the role of wild boars as intermediate hosts for *N. caninum* is due to its contribution to the circulation of the parasite in livestock. Concerning *S. scabiei* var. *suis*, it should be considered that the presence of positive wild boars near animal settling pose a sanitary risk and the use of serology should be proposed to estimate the mite infestation in wild boars.

Due to the rapid expansion of wild boars into urban and rural areas, the constitution of surveillance plans by using standardized methods should be emphasized, also to promote the awareness between hunters, wildlife professionals, and consumers, on the circulation of parasites of this wild species with an impact both on human and animal health. Therefore, further serological and molecular studies are needed for a better understanding of the epidemiology of these parasites in wildlife, to evaluate the effective risk for humans and livestock species, and to enforce monitoring and control programs.

## Figures and Tables

**Table 1:** Serological prevalence (P%) for *Toxoplasma gondii*, *Neospora caninum*, and *Sarcoptes scabiei* var. *suis* in 128 wild boars regularly hunted for human consumption in northern Italy.

Variable	Category	*Toxoplasma gondii*		*Neospora caninum*		*Sarcoptes scabiei* var. *suis*	
		***n* Positive**	**P%** **(95% CI)**	***n* Positive**	**P%** **(95% CI)**	***n* Positive**	**P%** **(95% CI)**
**Age**	**Young**	10	7.8 (31.6–12.5)	1	0.8 (0.74–2.31)	1	0.8 (0.74–2.31)
	**Adult**	31	24.2 (16.8–31.6)	5	3.9 (0.55–7.3)	6	4.7 (1.0–8.3)
	**Subadult**	27	21.1 (14.0–28.2)	8	6.2 (2.1–10.4)	2	1.6 (0.6–3.7)
**Sex**	**Male**	35	27.3 (19.6–35.1)	8	6.2 (2.1–10.4)	3	2.3 (0.3–4.9)
	**Female**	33	25.8 (18.2–33.4)	6	4.7 (1.0–8.3)	6	4.7 (1.0–8.3)
**Sampling** **season**	**Spring–** **Summer**	25	19.5 (12.7–26.4)	2	1.6 (0.6–3.7)	7	5.5 (1.5–9.4)
	**Autumn–Winter**	43	33.6 (25.4–41.8)	12	9.4 (4.3–14.4)	2	1.6 (0.6–3.7)
**Hunting** **municipality**	**Rural**	27	21.1 (14.0–28.2)	3	2.3 (0.3–4.9)	2	1.6 (0.6–3.7)
	**Intermediate**	41	32.0 (23.9–40.1)	11	8.6 (3.7–13.5)	7	5.5 (1.5–9.4)
**Overall**		68	53.1 (44.5–61.8)	14	10.9 (5.5–16.3)	9	7.0 (2.6–11.5)

95% CI: Confidence Interval.

**Table 2:** Results of the multivariate analysis of the risk factors related to *Sarcoptes scabiei* var. *suis* seroprevalence in wild boars in Lombardy, Italy.

Variable	Category	P%	β	Standard Error of Coefficients	Wald Chi- Square	Odds Ratio (95% Confidence Interval)	*p* Value	Akaike Information Criterion
**Hunting Season**	**Spring–Summer**	5.5	2.0	0.8	6.2	7.8 (1.5–39.1)	0.01	17.9
	**Autumn–Winter**	1.6	0			1		

## Data Availability

The datasets used and/or analyzed during the current study are available from the corresponding author on reasonable request.
